# The Effect of Course Length on Individual Medley Swimming Performance in National and International Athletes

**DOI:** 10.2478/hukin-2014-0073

**Published:** 2014-10-10

**Authors:** Mathias Wolfrum, Christoph Alexander Rüst, Thomas Rosemann, Romuald Lepers, Beat Knechtle

**Affiliations:** 1 Institute of Primary Care, University of Zurich, Zurich, Switzerland.; 2 Cardiovascular Center Cardiology, University Hospital Zürich, Zürich, Switzerland.; 3 INSERM U1093, Faculty of Sport Sciences, University of Burgundy, Dijon, France.; 4 Gesundheitszentrum St. Gallen, St. Gallen, Switzerland.

**Keywords:** swim speed, pool length, sex-related difference, temporal trends

## Abstract

Effects of course length (25 m versus 50 m) and advances in performance of individual medley swimming were examined for men and women in Swiss national competitions and FINA World Championships during 2000–2011. Linear regression and analysis of variance (ANOVA) were used to analyse 200 m and 400 m race results for 26,081 swims on the Swiss high score list and 382 FINA finalists. Swiss and FINA swimmers of both sexes were, on average, 4.3±3.2% faster on short courses for both race distances. Sex-related differences in swim speed were significantly greater for FINA swimmers competing in short-course events than in long-course events (10.3±0.2% versus 9.7±0.3%, p<0.01), but did not differ for Swiss swimmers (p>0.05). Sex-related differences in swimming speed decreased with increasing race distance for both short- and long-course events for Swiss athletes, and for FINA athletes in long-course events. Performance improved significantly (p<0.05) during 2000–2011 for FINA men competing in either course length and FINA females competing in short-course events, but not for Swiss swimmers. Overall, the results showed that men and women individual medley swimmers, competing at both national and international levels, have faster average swimming speeds on short courses than on long courses, for both 200 m and 400 m distances. FINA athletes demonstrate an improving performance in the vast majority of individual medley events, while performance at national level seems to have reached a plateau during 2000–2011.

## Introduction

Swimming competitions are held on both short (25 m) and long (50 m) courses (FINA, 2012a). Swim speeds are generally faster for short-course events (FINA, 2012b), due to the greater number of turns made for any given swim distance (Keskinen et al., 1996; Telford et al., 1988; Wakayosh et al., 1999). Turns provide increased propulsion and moderate exercise recuperation, resulting in several physiological and biomechanical differences between short and long course events such as a lower heart rate and blood lactate concentration in short course events of freestyle swimming (Keskinen et al., 2007; Lowensteyn et al., 1994; Telford et al., 1988). Effects of course length on swimming performance differ to some extent between men and women in freestyle swimming (Wirtz et al., 1992). Male freestyle swimmers gain more advantage from short-course events than their female counterparts, because men are able to reach higher swimming speeds during turns (Wirtz et al., 1992). However, no previous study has examined effects of course length on performance or sex-related differences in individual medley swimming.

A number of previous studies have used world records to investigate trends in performance over time in various Olympic disciplines (Berthelot et al., 2008; Lippi et al., 2008; Nevill and Whyte, 2005; Nevill et al., 2007). Top race times decreased during the last century in many endurance sport disciplines, including cycling, speed skating, triathlon, running, and swimming (Berthelot et al., 2008; Lepers, 2008), but reached plateaus in the 1970s in running (Berthelot et al., 2008; Lippi et al., 2008), swimming, and cycling (Berthelot et al., 2008). In younger sports disciplines such as triathlon the improvement plateaued in the 1990s (Lepers, 2008). Berthelot et al. (2008) suggested that the next generation would reach the physiological limit of human performance in sports, and predicted that half of all world records would improve only by 0.05% or less by 2027.

Temporal trends in swimming speed (Berthelot et al., 2010; Eichenberger et al., 2012; Eichenberger et al., 2013) and in sex-related differences (Eichenberger et al., 2012; Eichenberger et al., 2013; Nevill et al., 2007; Seiler et al., 2007; Thibault et al., 2010) have been investigated for freestyle (Eichenberger et al., 2012; Eichenberger et al., 2013; Nevill et al., 2007; Seiler et al., 2007) and backstroke (Seiler et al., 2007). These trends reflect the combined effects of improvements in performance and technology. Nevill et al. (2007) found accelerated improvement in freestyle swimming by men and women during the 1960s and 1970s, followed by a plateau during the 1980s and 1990s. However, world record times for a number of swim strokes improved by ∼2% during 2000 to 2010 (Berthelot et al., 2010), which was attributable to a combination of factors such as drag-reducing swimsuits (Berthelot et al., 2010), better pool designs, such as deeper pools and more effective ‘anti-wave’ lane ropes (Nevill et al., 2007), more efficient training approaches, including the use of the robotic underwater camcorder (Colwin, 2002), and better evaluation of swimmers, based on physiological variables, psychological skills, and emotional competency (Smith et al., 2002).

The present study examines male and female swimmers at national (annual Swiss high score list) and international (finalists in FINA World championships) level (i) individual medley swimming performance on short (25 m) versus long (50 m) courses for 200 m and 400 m swim races and (ii) temporal trends in individual medley swimming performance during the 2000–2011 period. Data for athletes competing at national level were available for each year whereas data for athlete competing at international level were available only every second year. We tested the hypotheses that (i) swimming speed would be faster on short course than on long course for both sexes and (ii) medley swimming performance would improve over time for both short- and long-course events.

## Material and Methods

Race results for all individual medley events on short (25 m) and long (50 m) courses were analysed for men and women on the annual Swiss high score list and finalists in the FINA World Swimming Championships during the 2000–2011 period. The data were obtained from the Swiss Swimming Federation (http://rankings.fsn.ch/) and from the Fédération Internationale de Natation (http://www.fina.org). This study was approved by the Institutional Review Board of St. Gallen, Switzerland, and the requirement for informed consent was waived, because the data are freely available to the public.

Data for Swiss athletes were available for a total of 13,093 swims (6,562 female swims and 6,531 male swims) competing in 200 m and 400 m short-course events, and for 12,988 swims (6,387 female swims and 6,601 male swims) in 200 m and 400 m long-course events. Data for 100 m races were excluded from the analysis, because they are held only on short courses. Data for FINA athletes competing in 200 m and 400 m races were available for a total of 191 swims (96 female swims and 95 male swims) in short-course events, and for 191 swims (95 female swims and 96 male swims) in long-course events.

Race times were converted to swimming speed prior to analyses using the equation [swimming speed in m/s] = [race distance in m] / [race time in s]. To examine temporal trends in performance and effects of course length, for Swiss swimmers the top ten swimming speeds for men and top ten swimming speeds for women were identified for races on long and short courses, for each race distance and year. The same analyses were performed for FINA athletes, using the top eight swimming speeds for male and female athletes. To investigate the difference between the performance trend among top FINA and Swiss athletes in comparison to the necessary annual improvement to gain a medal in consecutive championships, we analysed the performance trend of the third position (i.e. bronze medallist) over consecutive FINA World Championships during the study period.

The top ten swimming speeds for all twelve years of the respective event and course length were pooled to examine the interactive effect of sex and course length on swimming performance by Swiss and FINA athletes. Sex-related differences in swimming speed were calculated as the absolute value of ([swimming speed by women] – [swimming speed by men]) / [swimming speed by men] × 100. The sex-related difference was calculated for pairs of equally placed athletes (e.g., 1st place woman and man, 2nd place woman and man, etc.), and the mean sex-related difference (± SD) was calculated using paired data.

Prior to statistical analyses, each set of data was tested for normal distribution and homogeneity of variances. Normal distribution was tested using the D’Agostino and Pearson omnibus normality test; homogeneity of variances was tested using the Levene’s test in case of two groups, and the Bartlett’s test in case of more than two groups. The student’s t-test (with Welch’s correction in case of unequal variances) was used to determine the significance of differences between two groups. One-way ANOVA with subsequent Dunnett post-hoc analysis was used to determine the significance of differences between more than two groups. Two-way ANOVA was used to determine the significance of the interactive effect of gender and course length on performance. Linear regression was used to determine the significance of temporal changes in a variable. Interquartile ranges (IQR) (P75-P25) were calculated with the race times of both national and international swimmers to estimate the variability of the data within a group. Statistical analyses were performed using IBM SPSS Statistics (Version 19, IBM SPSS, Chicago, IL, USA) and GraphPad Prism (Version 5, GraphPad Software, La Jolla, CA, USA). Significance was accepted at p<0.05 (two-tailed for t-tests). Data reported in the text and figures are mean ± standard deviation (SD).

## Results

### Effects of course length on swimming speed and sex-related differences

**Figure 1:**
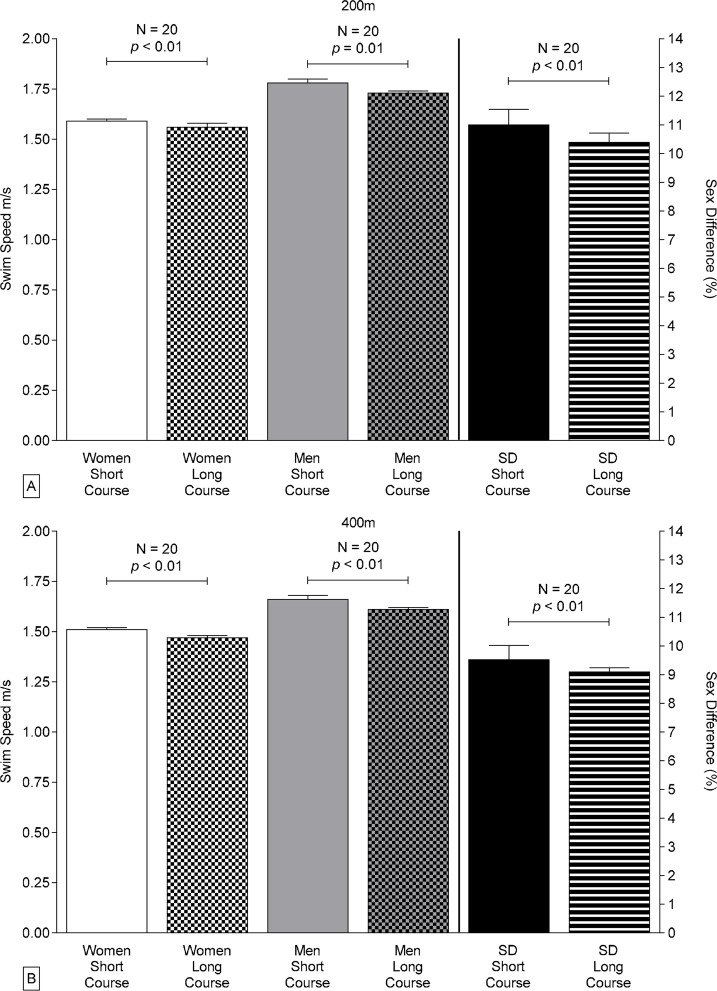
Swimming speed and sex related differences of the overall top ten FINA male and female individual medley swimmers between 2000 and 2011 over 200 m distance (Panel A) and 400 m distance (Panel B), respectively. The p-value is given in case of a significant difference between short course and long course swimming. “NS” indicates absence of a significant difference.

**Figure 2:**
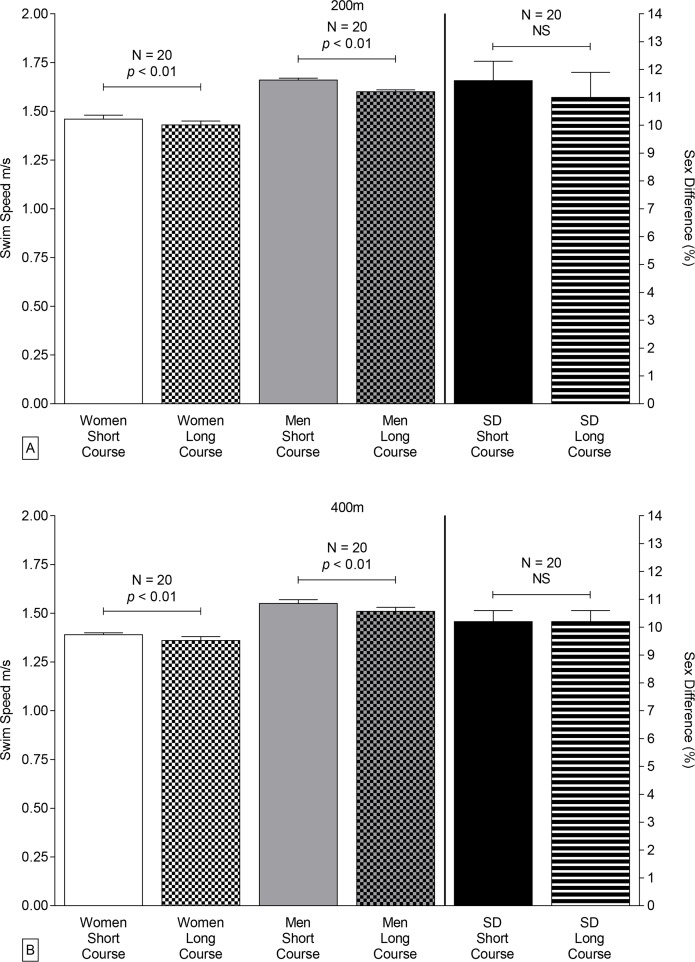
Swimming speed and sex related differences of the overall top ten Swiss male and female individual medley swimmers between 2000 and 2011 over 200 m distance (Panel A) and 400 m distance (Panel B), respectively. The p-value is given in case of a significant difference between short course and long course swimming. “NS” indicates absence of a significant difference.

Both men and women competing in 200 m and 400 m individual medley events at both national and international level were significantly faster on short courses than on long courses regardless of race distance (on average 4.3±3.2%, Figures 1 and 2, Tables 1 and 2).

Men had faster swimming speeds than women for all course lengths and distances (twoway ANOVA, *p*<0.0001). The interactive effect of sex and course length on swimming speed was significant for 200 m and 400 m at international level and for 400 m at national level (Table 2).

Sex-related differences in swimming speed were significantly greater in short-course than in long-course FINA events (on average 10.3±0.1% vs. 9.7±0.1%, *t*-test, *p*<0.0001, Figure 1). In contrast, the sex-related differences did not vary significantly with course length for Swiss swimmers (*p*>0.05). Sex-related differences in swimming speed of Swiss swimmers decreased with increasing race distance in both short- and long-course events, but only in long-course events for FINA swimmers (Table 3).

### Temporal changes in swim speed and sex-related differences

**Figure 3:**
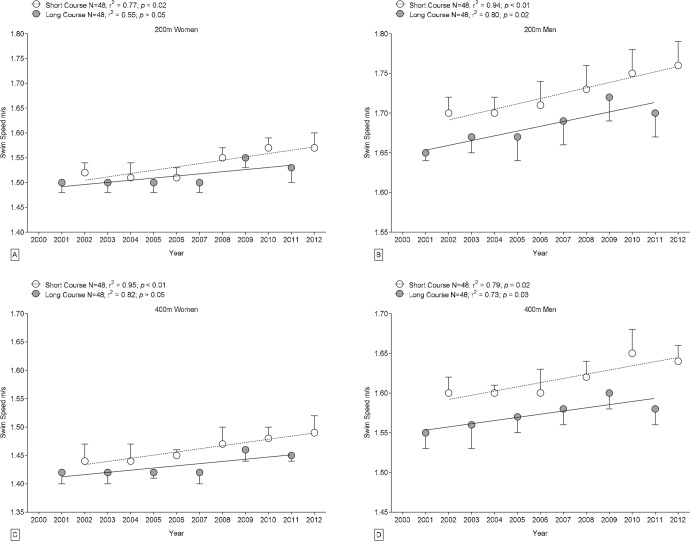
Changes in swimming speed of male and female FINA World Champion finalist individual medley swimmers during each year from 2000 to 2012 in short course versus long course events over 200 m and 400 m; Mean ± SD.

FINA swimmers showed significant improvement in performance during 2000–2011. Swimming speeds of FINA men competing in long-course events became faster in 200 m and 400 m races (0.006 m·s^−1^ *per annum* and 0.004 m·s^−1^ *per annum*, respectively, Figure 3) as well as in short course events in 200 m and 400 m races (0.007 m·s^−1^ *per annum* and 0.005 m·s^−1^ *per annum*, respectively). Furthermore, FINA female swimmers became faster in 200 m and 400 m races in short course events (0.007 m·s^−1^ *per annum* and 0.006 m·s^−1^ *per annum*, respectively).

**Figure 4:**
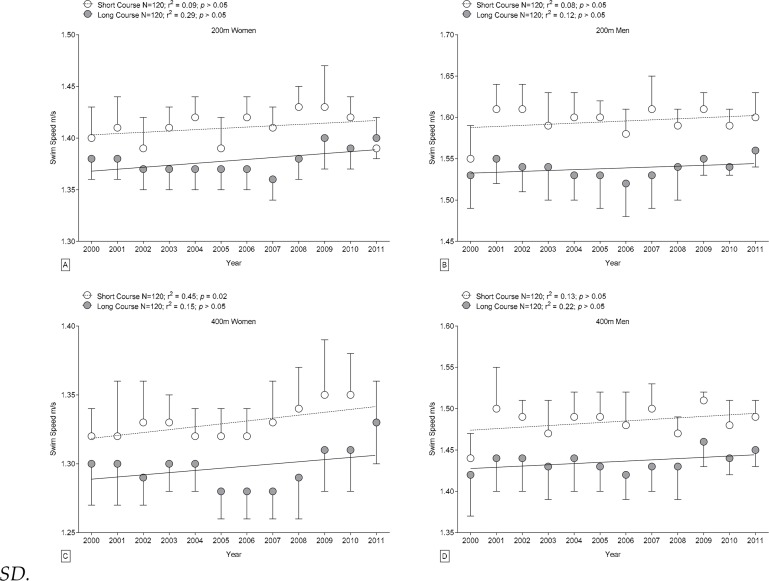
Changes in swimming speed of male and female Swiss individual medley swimmers during each year from 2000 to 2011 in short course versus long course events over 200 m and 400 m; Mean ± SD

Swimming speeds of Swiss women competing in 400 m short-course events also increased significantly (0.003 m·s^−1^ *per annum*) (Figure 4). However, FINA women competing in long-course events, Swiss men competing on either course length, and Swiss women competing in 200 m short-course events or in long-course events showed no significant improvement. The “clinical relevant improvement” of staying among the top three FINA swimmers to gain a medal during consecutive years is shown in Table 4. Athletes had to improve on average 0.005 m/s annually to gain a medal at consecutive FINA World Championships during the study period. Sex-related differences in swimming speed showed no significant changes over time.

## Discussion

The present study demonstrated that for FINA finalists and elite Swiss swimmers competing in 200 m and 400 m individual medley events (*i*) swimming speeds were significantly faster on short courses than on long courses for both men and women, (*ii*) sex-related differences in performance by FINA swimmers were significantly greater in short-course events than in long-course events, while sex-related differences in performance of Swiss athletes were not significantly affected by course length, (*iii*) sex-related differences in swimming speed decreased with increasing race distance, and (*iv*) swimming performance by FINA men competing in either course length and FINA females competing in 200 m and 400 m short-course events improved during 2000–2011.

### Effects of course length on swimming speed

The finding that female and male individually medley swimmers were faster on short (25 m) than on long (50 m) courses is broadly recognized in swimming, and confirms results of previous studies of freestyle swimming (Keskinen et al., 2007; Lowensteyn et al., 1994). During 200 m freestyle events, swimmers spend about twice as long turning and gliding in the short-course pool than in the long-course pool (Keskinen et al., 2007). Swimmers with good turning ability and high potential for muscular force production benefit from swimming on short courses, because each turn offers time for muscle recovery leading to decreased lactate production and increased lactate clearance by the upper body and arm muscles used for regular stroking (Craig, 1986; Keskinen et al., 2007; Lowensteyn et al., 1994; Telford et al., 1988; Wirtz et al., 1992). Furthermore, some elite swimmers demonstrated a higher stroke frequency and a larger distance covered per stroke during short-course events than during long-course events (Wirtz et al., 1992), which leads to higher swimming performance (Craig et al., 1985).

### Effects of course length on sex-related differences in performance

In freestyle swimming, men gain more advantage than women from turning and, therefore, sex-related differences in performance are generally greater in short-course than in long-course events (Wirtz et al., 1992). In the present study, we found a similar pattern for individual medley performance by FINA swimmers on either course length. Swiss swimmers demonstrated a trend towards a greater sex-related difference in performance in 200 m races in short course events versus long course events.

In general trends in individual medley swimming are more difficult to explain than trends in freestyle swimming, because individual medley consists of four swimming styles in each race, and there is conflicting evidence regarding the most important stroke. One study found that backstroke was most closely correlated with performance (Saavedra et al., 2012); another study concluded that breaststroke was most important (Mason and Cossor, 2000). The lack of significant sex-related differences in speed of Swiss swimmers in the present study might be attributable to a combination of two factors affecting performance at intermediate (200 m and 400 m) race distances: sex-related differences in freestyle swimming speed are enhanced by increasing race distance, as men gain more advantage from a greater number of turns, but at the same time, sex-related differences are reduced by increasing race distance, due to the higher economy of female swimmers (Hinrichs, 2007; Lavoie and Montpetit, 1986; Pendergast et al., 1977; Toussaint et al., 1988).

### Temporal changes in swim speed and sex-related differences

Temporal trends in athletic performance are attributable to several factors such as technological advances (Berthelot et al., 2010), improved techniques and/or training protocols (Issurin, 2010), changes in anthropometric and physiological variables (Lätt and Jürimaë, 2010), and changes in psychological (Psychountaki and Zervas, 2000) and nutritional aspects (Stellingwerff et al., 2011).

Our hypothesis that individual medley performance would improve over time was confirmed for male FINA finalists competing in long- and short course events and for female FINA finalists competing in short course events. FINA female competitors in long-course events and Swiss swimmers showed no significant changes in swimming performance during 2000–2011. Our findings in athletes at international level are in line with studies of individual medley long-course events during earlier periods that found improvements in swimming performance (Berthelot et al., 2010; Buhl et al., 2013). The general lack of significant improvement in Swiss swimmers during 2000–2011 could be at least partly due to the relatively short time span of the analysis. During that period, furthermore, technological advances in swim suits were introduced, and record swimming performances were achieved, but FINA regulated the use of full-body, polyurethane swimsuits in 2009, and the Swiss Swimming Federation followed suit. Following the regulation of swim suits, no Swiss national record in individual medley swimming was broken, with a single exception for men’s 200 m in 2012 (Schweizer-Schwimmverband, 2012).

The finding of improved performance over men’s FINA long course events might be at least attributed to the prestige of this course length. Long course events are held during the Olympic Games, an event that is of paramount importance to every professional athlete in light of not only personal and social but also monetary rewards. With regard to the Olympic Games, elite swimmers train in particular long course races, thereby enhancing their performance especially over that particular pool length.

The general lack of improvement in Swiss individual medley swimming might reflect the fact that Swiss swimmers in contrast to international swimmers are selected from a smaller pool of athletes, make less use of advanced technologies, or undergo less intense training (Mujika, 1998; Mujika et al., 1995). Swiss swimmers might also be less subjected to sports psychology and motivation, *i.e*., less focused on success in competition than competitors at international level (Johnson et al., 2009; Miller, 1993). Another explanation for the lack of improving performance in Swiss medley swimmers might be the focus of interest. To our knowledge, there are only a few Swiss elite swimmers who specialize in medley swimming and are able to compete successfully at international level. Out of this limited pool, only one man was able to qualify for a semi-final at a FINA World Swimming Championship (*i.e.* Lorenz Liechti, 100 m medley semi-final, Indianapolis (USA), 2004). Furthermore, this fact also explains the relatively large difference between the current world records and the Swiss records in medley swimming which is for 200 m about 7 s and for 400 m about 17 s. On the other hand, there are numerous Swiss athletes who are able to compete successfully at FINA World Championships on a regular basis by specializing in a specific stroke, e.g. Flori Lang (50 m backstroke) and Dominik Meichtry (200 m freestyle).

Another important point is the limited pool of upcoming youth Swiss swimmers compared to other well-known swimming -nations like the United States, Russia or China. Because of its alpine conditions Switzerland is most famous in sports like alpine skiing, ice-hockey, ski-jumping, etc. Indeed, Switzerland is holding the 7^th^ position among the top-ten nations that joined the Winter Olympic Games but is only on the 17^th^ position among nations that joined the Summer Olympic Games according to the all-time medallist table for the Olympic Games (Olympics 2013).

Interestingly, there is a huge gap between the observed statistical trend of individual medley swimming performance among the FINA finalists and the “clinical relevant improvement” of staying among the top three FINA swimmers to gain a medal during consecutive years. Depending on the course length and the race distance and based on the performance of the top three FINA swimmers one had to improve approximately 0.005 m/s annually to gain a medal at consecutive FINA World Championships during the study period. This increase in performance was only fulfilled by FINA male individual medley finalists at long course events suggesting rather low variability among this group of top athletes participating in this highly popular Olympic event. Looking at the performance trend of FINA and Swiss athletes of the remaining events reveals a more heterogeneous result. Even among FINA World Championships finalists only a small number of exceptional athletes is able to raise the performance level over years, while the remaining athletes fail. Nowadays, top performance is limited to a small pool of athletes that can provide top performance in consecutive years. One of them is Michael Phelps who was able to stay among the top three individual medley swimmers at consecutive FINA World Championships over the study period (FINA, 2012b).

Sex-related differences in individual medley performance did not change significantly for FINA or Swiss medley swimmers during 2000–2011. This result confirms that of Nevill and colleagues, who reported that sex-related differences in various swimming and running events were remarkably stable, at ∼10%, during the past 60 years (Nevill et al., 2007). Other studies that examined performance in freestyle swimming during the last century (Schulz and Curnow, 1988) and the last two decades (O'Connor and Vozenilek, 2011) came to the same conclusion. As the factors that influence sex-related differences in swimming performance, *i.e.* body dimensions, buoyancy, stroke mechanics, anaerobic power and capacity, muscle power, flexibility, start and turn times, and basic and specific endurance, probably do not change rapidly (Nevill et al., 2007; Smith et al., 2002; Tanaka and Seals, 1997), a significant change in sex-related differences in swim performance during an 11-year period is unlikely.

A limitation of this study is that we compared annual data of swimmers at national level of the annual high score list to biannual data of finalists in the World Championships. The statistical difference depends on the variability in a group and the number of investigated subjects within a group. A higher level group such as FINA swimmers may have lower variability when compared to a lower level group such as Swiss-swimmers. We, therefore, calculated the IQR for each group and compared between the levels (Table 5). The value for P75-P25 was lower in Swiss swimmers compared to FINA swimmers for both courses in both women and men. This might, however, show that the data of the Swiss swimmers had a lower variability level than the data of the FINA swimmers.

## Conclusion

Although swimming speeds on short (25 m) courses compared to speeds on long (50 m) courses are widely acknowledged in swimming, the present study is the first to demonstrate this course-length effect in individual medley swimming, and the effect held true for men and women competing in 200 m and 400 m races in Swiss and international competitions. Swiss and FINA swimmers were on average 4.3±3.2% faster on short courses than on long courses. FINA competitors in individual medley events demonstrated a greater sex-related difference in performance in short-course pools compared to long course pools. Only FINA men competing in either course length and FINA females competing in short-course events consistently improved swimming performance during the 11-year study period. However, performance of Swiss swimmers was consistently slower than that of FINA finalists, suggesting that Swiss swimmers might benefit from more vigorous and optimized training programs, focused on muscular force production in combination with efficient swimming skills. Further research, especially including effects of anthropometric, biomechanical, and physiological factors, is required to fully understand the course-length effect on individual medley swimming. Future research should focus not only on overall performance, but on performance in each of the four swimming styles used in individual medley competitions.

## Figures and Tables

**Table 1:** Difference (%) in swim speed

	200 m	400 m	
**Men**			

FINA	1.69 ± 0.85	2.53 ± 1.05	*p* = 0.006
Swiss	3.41 ± 0.14	2.65 ± 0.73	*p* = 0.009

**Women**			

FINA	9.72 ± 3.52	8.96 ± 2.93	*p* = 0.18
Swiss	2.71 ± 0.72	2.59 ± 0.65	*p* = 0.90

Differences in swim speeds on short (25 m) and long (50 m) courses of the overall top ten FINA and of the overall top ten Swiss individual medley swimmers between 2000 and 2011. Values are mean ± SD. N = 80.

**Table 2:** Results of 2-way ANOVA

	sex	course length	sex × course length
**FINA** **Swim distance**			

200 m	*F* = 285.8, *p* < 0.0001	*F* = 14.9, *p* = 0.0002	*F* = 7.4, *p* = 0.007
400 m	*F* = 308.5, p < 0.0001	*F* = 28.4, p < 0.0001	*F* = 6.2, p = 0.014

**Swiss** **Swim distance**			

200 m	*F* = 4474.8, *p* < 0.0001	*F* = 247.7, *p* < 0.0001	*F* = 15.5, *p* < 0.0001
400 m	*F* = 2803.3, p < 0.0001	*F* = 163.3, p < 0.0001	*F* = 3.3, p = 0.069

Statistical significance (2-way ANOVA) of effects of sex and course length and the interactive effects of sex × course length on speed of the overall top ten FINA and of the overall top ten Swiss individual medley swimmers between 2000 and 2011. N = 80.

**Table 3:** Swim speed (m/s) to gain 3-rd position in FINA World Championship finals

	2001/ 2002	2011/ 2012	average annual improvement (m/s)
**FINA - Men**			

short course			
200 m	1.70	1.77	0.007 (4.1%)
400 m	1.61	1.65	0.004 (2.8%)
long course			
200 m	1.65	1.70	0.005 (3.0%)
400 m	1.56	1.59	0.003 (1.9%)

**FINA - Women**			

short course			
200 m	1.52	1.57	0.005 (3.3%)
400 m	1.45	1.50	0.005 (3.4%)
long course			
200 m	1.51	1.55	0.004 (2.6%)
400 m	1.43	1.46	0.003 (2.1%)

Sex differences in swim speeds in short (25 m) and long (50 m) courses of the overall top ten FINA and of the overall top ten Swiss individual medley swimmers between 2000 and 2011. Values are mean ± SD. N = 80.

**Table 4:** Sex difference in swim speed (%)

	200 m	400 m	
**FINA**			

short course	2.4 ± 3.6	2.8 ± 2.8	*p* > 0.05
long course	11.6 ± 0.4	10.0 ± 0.2	*p* < 0.05

**Swiss**			

short course	11.6 ± 0.7	10.2 ± 0.4	*p* < 0.05
long course	11.0 ± 0.9	10.2 ± 0.4	*p* < 0.05

Swim speed to gain a medal (based on the bronze medallist) in FINA World Championships during 2001–20012 with the corresponding annual improvement of swim speed to achieve this performance level. Values are in (m/s).

**Table 5:** 

**Distance**	**Men**			**Women**		

	**FINA**	**Swiss**	**Δ**	**FINA**	**Swiss**	**Δ**
Short course 200 m	0.067	0.02	0.047	0.108	0.027	0.081
Short course 400 m	0.055	0.025	0.03	0.08	0.018	0.062

Mean ± SD	0.061±0.0084	0.022±0.0035	0.038±0.012	0.094±0.019	0.022±0.0063	0.071±0.013

Long course 200 m	0.04	0.018	0.022	0.035	0.018	0.017
Long course 400 m	0.027	0.01	0.017	0.033	0.025	0.008

Mean ± SD	0.033±0.0091	0.014±0.0056	0.019±0.0035	0.034±0.0014	0.021±0.0049	0.012±0.0063

IQR (P75-P25) for FINA and Swiss swimmers for each distance and discipline including the difference between the levels.

Mean ± SD are presented for the IQR within a group (i.e. male FINA Swimmers, male Swiss swimmers, etc)
